# A latent class analysis of cardiometabolic risk factors and the predicted prevalence of subclinical atherosclerosis in middle-aged Swedish adults

**DOI:** 10.1038/s41598-026-42858-5

**Published:** 2026-03-04

**Authors:** Kanya Anindya, Marcus Bendtsen, Tomas Jernberg, Susanna Calling, Lars Lind, Lars Weinehall, Nawi Ng, Maria Rosvall

**Affiliations:** 1 School of Public Health and Community Medicine, Institute of Medicine, Sahlgrenska Academy, University of Gothenburg, Guldhedsgatan 5A, plan 3, Gothenburg, 413 20 Sweden; 2 Department of Health, Medicine and Caring Sciences, Linköping University, Linköping, Sweden; 3 Department of Clinical Sciences, Danderyd Hospital, Karolinska Institute, Stockholm, Sweden; 4 Center for Primary Health Care Research, Department of Clinical Sciences Malmö, Lund University, Malmö, Sweden; 5 Office for Primary Care, Skåne University Hospital, Lund, Sweden; 6 Department of Medical Sciences, Uppsala University, Uppsala, Sweden; 7 Department of Epidemiology and Global Health, Umeå University, Umeå, Sweden; 8 Social Medicine, Region Västra Götaland, FoUUI, Regionhälsan, Sweden

**Keywords:** Atherosclerosis, Cardiovascular disease, Cardiometabolic risk factors, Latent class analysis, Bias-adjusted three-step estimation, Biomarkers, Cardiology, Diseases, Endocrinology, Health care, Medical research, Risk factors

## Abstract

Previous research on cardiometabolic risk has mostly used a variable-centred approach, assessing risk factors separately or in predefined combinations. This study used a probabilistic modelling approach to identify distinct cardiometabolic risk classes and estimate the predicted prevalence of subclinical atherosclerosis. The analysis included 28,307 middle-aged adults from the Swedish CArdioPulmonary bioImage Study (2013–2018), linked to national registers. Eleven risk factors were assessed: smoking, alcohol consumption, sodium and fibre intake, physical activity, stress, waist circumference, triglycerides, HDL-cholesterol, blood pressure, and fasting glucose. Subclinical atherosclerosis was defined using coronary artery calcium (CAC) scores and the presence of carotid plaque. A three-step latent class analysis identified four cardiometabolic risk classes: “low fibre intake and normolipidemia” (55.2%, Class 1), “high sodium intake and normolipidemia” (12.8%, Class 2), “unhealthy lifestyle and heightened metabolic risk” (10.1%, Class 3), and “unhealthy lifestyle and high metabolic risk” (21.9%, Class 4). Predicted mean CAC scores ranged from 42.6 (Class 2, 95% CI 39.0–46.3) to 92.1 (Class 4, 95% CI 86.2–98.0). Predicted carotid plaque prevalence ranged from 51.6% (Class 2, 95% CI 50.6–52.6) to 60.8% (Class 4, 95% CI 59.8–61.9). Latent classes offered a complementary descriptive framework beyond single risk factors, supporting more tailored prevention according to risk profiles.

**Supplementary Information:**

The online version contains supplementary material available at 10.1038/s41598-026-42858-5.

## Introduction

Atherosclerosis, the presence of plaque formation and inflammation in the arterial wall, is the underlying pathology of most cardiovascular disease (CVD).^1^ Atherosclerotic lesions develop gradually before clinical symptoms occur, a phase known as subclinical atherosclerosis.^1^ The prevalence of subclinical atherosclerosis is substantial, where 42.1% of adults aged 50–64 years without a history of coronary heart disease in Sweden show some degree of coronary atherosclerosis.^2^ This silent period is a critical window for intervention, as early management of risk factors can prevent progression to major CVD events.

Metabolic risk factors, including abdominal obesity, hypertension, dyslipidemia, and dysglycaemia, are involved in the pathogenesis of atherosclerotic cardiovascular diseases (ASCVD).^3–6^ Prior studies also highlight that behavioural risk factors (i.e., smoking, alcohol consumption, unhealthy diets, sedentary lifestyle) and psychosocial stress are associated with metabolic risk factors and ASCVD.^7,8^ Previous evidence shows that the co-occurrence of risk factors in a person is associated with a greater risk of ASCVD.^9–12^.

While the importance of risk factor clustering is well documented, previous research has often relied on a ‘variable-centred’ approach, such as predefining specific combinations of risk factors (e.g., smoking, hypertension, dyslipidemia, overweight).^9,10,12^ These approaches remain widely used because they are simple, interpretable, and align closely with clinical guidelines that target individual risk factors. Such an approach has conceptual and practical limitations due to the exponential growth in the number of possible combinations (2^k^) as the number of risk factors (k) increases, leading to data sparsity and unstable estimates.^13^ As a result, studies using this approach have been constrained to a limited number of combinations that are deemed to be ‘clinically important’, potentially overlooking higher-order interactions of risk factors that are important to prevent ASCVD.^13^ Accordingly, latent class analysis (LCA), a probabilistic modelling approach,^14^ can be used to identify common constellations of risk factors without pre-specifying combinations, thereby providing complementary information to traditional variable-centred approaches.

LCA identifies unobserved (‘latent’) patterns of risk factors within the data using observed categorical indicators.^13–15^ LCA identifies a parsimonious set of risk factor classes, where each class is assumed to be homogeneous while being heterogeneous across classes.^14,15^ Thus, the novelty of applying LCA in this context is its ability to reveal complex co-occurrence patterns of risk factors, while accounting for uncertainty in class assignment through probabilistic membership, which may be associated with atherosclerosis.^13^ Using LCA, this study aims to (i) identify the latent classes of cardiometabolic risk factors, and (ii) estimate the predicted prevalence of subclinical coronary and carotid atherosclerosis between latent classes in middle-aged Swedish adults.

## Methods

### Data and population

This cross-sectional study used the Swedish CArdioPulmonary bioImage Study (SCAPIS) database (Petition-447).^16^ SCAPIS is a prospective population-based study that randomly recruited 30,154 individuals aged 50–64 years living in six areas surrounding Swedish university hospital (Gothenburg, Linköping, Malmö/Lund, Stockholm, Umeå and Uppsala) from November 2013 to December 2018.^16^ The dataset was linked to the Swedish Prescribed Drug Register and the National Patient Register for inpatient care to identify participants’ history of CVD. The exclusion criteria of this study were participants who did not consent to link their responses in SCAPIS to the register data (*n* = 124), had not performed computed tomography (*n* = 541), or had missing data on coronary and carotid atherosclerosis (*n* = 250). Participants who had a history of coronary heart disease, stroke, or percutaneous coronary intervention/coronary artery bypass grafting (PCI/CABG) (*n* = 932) were also excluded, as the aim of the study is to observe subclinical atherosclerosis. Sample flowcharts and ICD codes used for exclusion criteria are available in Figure S1. A total of 28,307 participants were considered in the main analysis.

SCAPIS study received ethical approval from the Regional Ethical Reviewer Board in Umeå (Dnr 2010-228-31 M). Written informed consent was obtained from all participants, and the study was carried out in accordance with the principles of the Declaration of Helsinki. This study was approved by the Swedish Ethical Review Board (Dnr 2022-03205-01) on 2022-06-29.

### Variables

#### Cardiometabolic risk factors (indicator variables)

We considered 11 indicator variables to examine profiles of cardiometabolic risk factors, including behavioural (i.e., smoking, high alcohol consumption, high sodium and low fibre intakes, and physical inactivity), psychosocial stress, and metabolic risk factors (i.e., elevated waist circumference, elevated triglycerides, reduced HDL-cholesterol, elevated blood pressure, and elevated fasting glucose).^8,17^.

Smoking was assessed based on self-reported history of smoking (“Do you smoke?“). Participants were categorised into non-smokers, former smokers, or current smokers.^18^ High alcohol consumption was defined using the first three Alcohol Use Disorders Identification Test (AUDIT) questions: the frequency of having an alcoholic drink, the number of alcoholic drinks on a typical drinking day, and the frequency of having > 6 alcoholic drinks on one occasion.^19^ Participants who consumed > 6 units per week or > 6 units on one occasion were categorised as having high alcohol consumption (Table S1).^20,21^.

Fibre and sodium intakes were assessed based on calculated nutrition values from the food frequency questionnaire. Participants who consumed < 25 g/day of fibre for females, or < 35 g/day of fibre for males, were classified as having low fibre intake.^22^ High sodium intake was defined as consuming sodium > 2.3 g/day.^22^ Physical activity intensity was measured using an accelerometer recording.^23^ Those with ≥ 9.5 h of sedentary activity per day and < 150 min of vigorous physical activity per week were classified as physically inactive (Table S1).^23^ Psychosocial stress was identified as persistent feelings of tension, irritability, anxiety, or difficulty sleeping due to work or home conditions. Participants who reported constant stress in the past year or longer were considered to have psychosocial stress.^24^.

Metabolic risk factors were assessed based on blood sampling analysis and a history of prescribed drugs in the last sixty months preceding the time of the interview (Table S1, the Anatomical Therapeutic Chemical/ATC codes). The National Cholesterol Education Program’s (NCEP) Adult Treatment Panel III (ATP III) was used as the guideline to determine the presence of metabolic risk factors. The guideline has been widely used in epidemiological studies and shows high sensitivity and specificity in identifying metabolic syndrome (MetS) associated with ASCVD.^25^ ATP III includes five components: (1) elevated waist circumference (> 102 for males or > 88 cm for females); (2) elevated blood pressure (systolic blood pressure > 130 mmHg and/or diastolic blood pressure > 85 mmHg or on anti-hypertensive medication; 3) elevated triglycerides (≥ 150 mg/dl or on lipid-lowering medication); 4) reduced HDL-cholesterol (≤ 45 mg/dl for males or ≤ 55 mg/dl for females or on lipid-lowering medication); and 5) elevated fasting glucose (≥ 100 mg/dl or on antidiabetic medication).^26^ Waist circumference was chosen over BMI to align with ATP III and expert panel recommendations because it better captures abdominal/visceral adiposity and reduces body mass index (BMI)-related misclassification of cardiometabolic and CVD risk.^27^ Binary variables were generated for each metabolic risk factor (no/yes). Individuals were classified as having MetS if they met three or more of the five components.^26^.

#### Subclinical atherosclerosis (outcome)

Subclinical coronary atherosclerosis was examined using coronary artery calcium (CAC) imaging with non-contrast electrocardiogram-gated computed tomography (CT) scanning (Siemens, Somatom Definition Flash, Siemens Healthineers, Erlangen, Germany).^2,16^ The Agatston scoring method was used to calculate the total calcium content in the left main, left anterior descending, circumflex, and right coronary arteries. Details of the CAC imaging procedure in SCAPIS have been documented in previous studies.^2,16^ The CAC score was classified into no/mild (< 100) or moderate/high (≥ 100).^16,28^ The CAC score was also measured as a continuous variable.

Subclinical carotid atherosclerosis was assessed using two-dimensional grayscale images of the common carotid artery (CCA), carotid bulb, and internal carotid artery (ICA) on both the left and right sides, performed with Siemens Acuson S2000 ultrasound scanner equipped with a 9L4 linear transducer.^2,16,28^ The images were evaluated to determine the intima-media thickness, size, and number of plaques. Following the Mannheim consensus, a carotid plaque was defined as “a focal structure that encroaches into the arterial lumen of at least 0.5 mm or 50% of the surrounding intima-media thickness value or demonstrates a thickness > 1.5 mm as measured from the media-adventitia interface to the intima-lumen interface”.^29^ Detailed measurements of carotid artery plaque have been covered elsewhere.^2,16,28^ Carotid atherosclerosis was classified as absent (no plaque in either carotid artery), unilateral (plaque in one carotid artery), and bilateral (plaques in both carotid arteries).

#### Sociodemographic characteristics (covariates)

The sociodemographic variables included age (in years), sex (females, males), marital status (married/cohabitation, single, divorced/widow), family history of diabetes or CVD (no, yes), country of birth (Swedish-born, foreign-born), educational attainment (tertiary, secondary, primary/lower), employment (employed, unemployed), and residency type (urban/suburban, rural) based on Demographic Statistical Areas (DeSO) (Table S1).

### Statistical analyses

We employed a bias-adjusted three-step LCA to identify subgroups of individuals with similar cardiometabolic profiles and to examine their association with subclinical atherosclerosis as a distal outcome. LCA was chosen over other approaches, such as standard cluster analysis, as it provides posterior membership probabilities. These probabilities allow the quantification of classification error, which can be subsequently incorporated into the estimation of associations between latent classes and distal outcomes to account for misclassification bias.^30^.

The analysis was conducted in three steps. *First*, we included 11 cardiometabolic risk factors in the latent class model. Covariates and outcomes were excluded in the first step to avoid class formation bias. Missing data on indicator variables were handled using full information maximum likelihood (FIML).^14^ We compared models with one to six classes using the Akaike Information Criterion (AIC), consistent AIC (CAIC), Bayesian Information Criterion (BIC), sample-size-adjusted BIC (SABIC), and entropy.^31^ The optimum number of classes was determined by balancing statistical fit with interpretability.^31^ To address violations of the local independence assumption, we included direct effects (pairwise associations) between indicators with bivariate residuals (BVRs) higher than four.

*Second*, each participant was assigned to the class with the highest probability of membership. Posterior class probabilities and proportional Bolck–Croon–Hagenaars (BCH) weights were retained to reduce classification error in the subsequent analyses.^30^ Compared with alternative approaches, we used the BCH method because it adjusts for classification error while keeping the latent class solution fixed, avoiding shifts in class definition when distal outcomes are added.^32^ BCH is also recommended for distal outcome analyses with both continuous and categorical outcomes.^33,34^.

*Third*, associations between latent classes and subclinical atherosclerosis were estimated using three different regression models, depending on the type of outcome variables: (1) binary logistic regression for the binary CAC score (< 100 or ≥ 100); (2) zero-inflated negative binomial regression for count CAC scores; and (3) multinomial logistic regression for carotid plaques (absent, unilateral, bilateral).^28^ Model fit was assessed using model-specific diagnostics: AUC and calibration for binary logistic models, information criteria and evaluation of excess zeros and overdispersion for zero-inflated negative binomial models, and likelihood-based fit indices with one vs. rest calibration of predicted category probabilities for multinomial logistic models. We reported covariate-adjusted predicted prevalence and predicted scores for each class, as these provide clinically interpretable estimates of burden rather than relative measures (e.g., odds ratio/OR). Missing data on covariates were handled using multiple imputations by chained equations with 20 repetitions.^35^.

Lastly, four sensitivity analyses were conducted. First, we performed an LCA stratified by sex (females and males) to qualitatively assess the stability and structure of the latent classes across groups. Second, a latent class model with elevated LDL-cholesterol (≥ 130 mg/dl or prescribed by lipid-lowering drugs), instead of elevated triglycerides, was constructed to compare the model fit with the main model. Third, we fitted an alternative mixture model incorporating continuous measures of alcohol intake (units/week), sodium intake (g/day), and fibre intake (g/day), with these variables standardised prior to modelling. Fourth, we assessed effect modification by sex by including a latent class × sex interaction term in the regression models. Steps 1 and 2 of the LCA were performed using LatentGOLD 6.1 (Statistical Innovations Inc., Belmont, MA, USA), while Step 3 was performed in Stata 18.0 SE (StataCorp LLC, College Station, TX, USA).

## Results

The mean age of study participants was 57.0 years (standard deviation/SD = 4.3) (Table 1). Of the 28,307 participants, 52.0% were female. 40.5% of participants had a CAC score greater than zero, with a median score of 36 (interquartile range/IQR 8–128). The prevalence of unilateral and bilateral plaque was 29.9% and 24.6%, respectively.

**Table 1:** Participants’ characteristics.

Variables	Total
N (%^a^)	28,307 (100)
Age, mean (SD)	57.0 (4.3)
Sex	
Female	14,732 (52.0)
Male	13,575 (48.0)
Marital status	
Single	3,694 (13.0)
Married	20,333 (71.8)
Divorce/widow	3,538 (12.5)
Missing	742 (2.6)
Country of birth	
Swedish-born	23,248 (82.1)
Foreign-born	4,391 (15.5)
Missing	668 (2.4)
Parental history of CVD	
No	12,737 (45.0)
Yes	14,562 (51.4)
Missing	1,008 (3.6)
Education	
Tertiary	12,589 (44.5)
Secondary	12,539 (44.3)
Primary or lower	2,482 (8.8)
Missing	697 (2.5)
Employment status	
Employed	24,156 (85.3)
Unemployed	3,355 (11.9)
Missing	796 (2.8)
Site	
Göteborg	5,903 (20.9)
Linköping	4,791 (16.9)
Malmö	5,820 (20.6)
Stockholm	4,713 (16.6)
Umeå	2,367 (8.4)
Uppsala	4,713 (16.6)
CAC score	
0	16,850 (59.5)
1–99	8,122 (28.7)
100–399	2,320 (8.2)
≥400	1,015 (3.6)
CAC score, median (IQR)^a^	36 (8–128)
Carotid artery plaques	
No plaque	12,880 (45.5)
Unilateral plaque	8,458 (29.9)
Bilateral plaque	6,969 (24.6)

CAC score: coronary artery calcium score, CVD: cardiovascular disease, SD: standard deviation, IQR: interquartile range. ^a^ Median CAC score was calculated only for participants with a CAC score > 0.

**Fig. 1:**
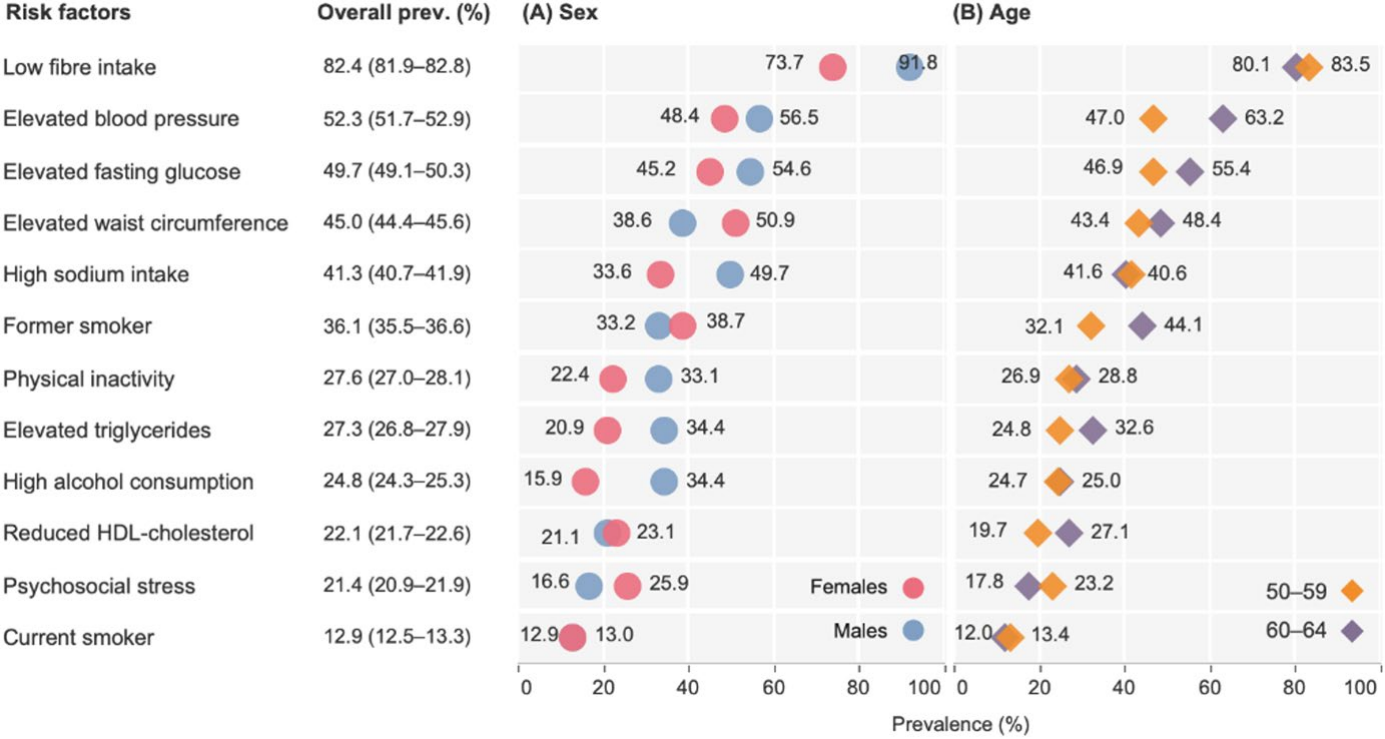
Prevalence of cardiometabolic risk factors in middle-aged Swedish adults, by sex and age groups. Risk factors are sorted based on the overall prevalence, from highest to lowest. Prevalences used multiple imputation (m = 20) for missing values, pooled with Rubin’s rules.

Figure 1 shows the prevalence of cardiometabolic risk factors by sex and age group. The most prevalent behavioural risk factors were low fibre intake (82.4%) and high sodium intake (41.3%), with a higher prevalence in males than females. Physical inactivity was reported by 27.6%, and 21.4% reported psychosocial stress, with males being more inactive and females reporting more stress. 36.1% and 12.9% of participants were former and current smokers, respectively, with former smokers slightly higher among males. High alcohol consumption was observed in 24.8% of the population, with males reporting more than two times higher prevalence than females. The prevalence of behavioural and psychosocial risk factors showed subtle differences across age groups, except for former smoking.

The three most common metabolic risk factors were elevated blood pressure (52.3%), elevated fasting glucose (49.7%), and elevated waist circumference (45.0%). Males generally had a greater prevalence of metabolic risk factors, except for elevated waist circumference and reduced HDL-cholesterol. The prevalence of metabolic risk factors was consistently higher in individuals aged 60–64 years.

### Latent class of cardiometabolic risk factor pattern

Based on model fit assessment, the four-class model was identified as the best fit, with the lowest BIC and SABIC values and good class separation (entropy = 0.81) (Table S3). All four classes were distinct and clinically interpretable, with class-specific mean posterior membership probabilities ranging from 0.71 (Class 3) to 0.95 (Class 1) (Table S4).

**Fig. 2:**
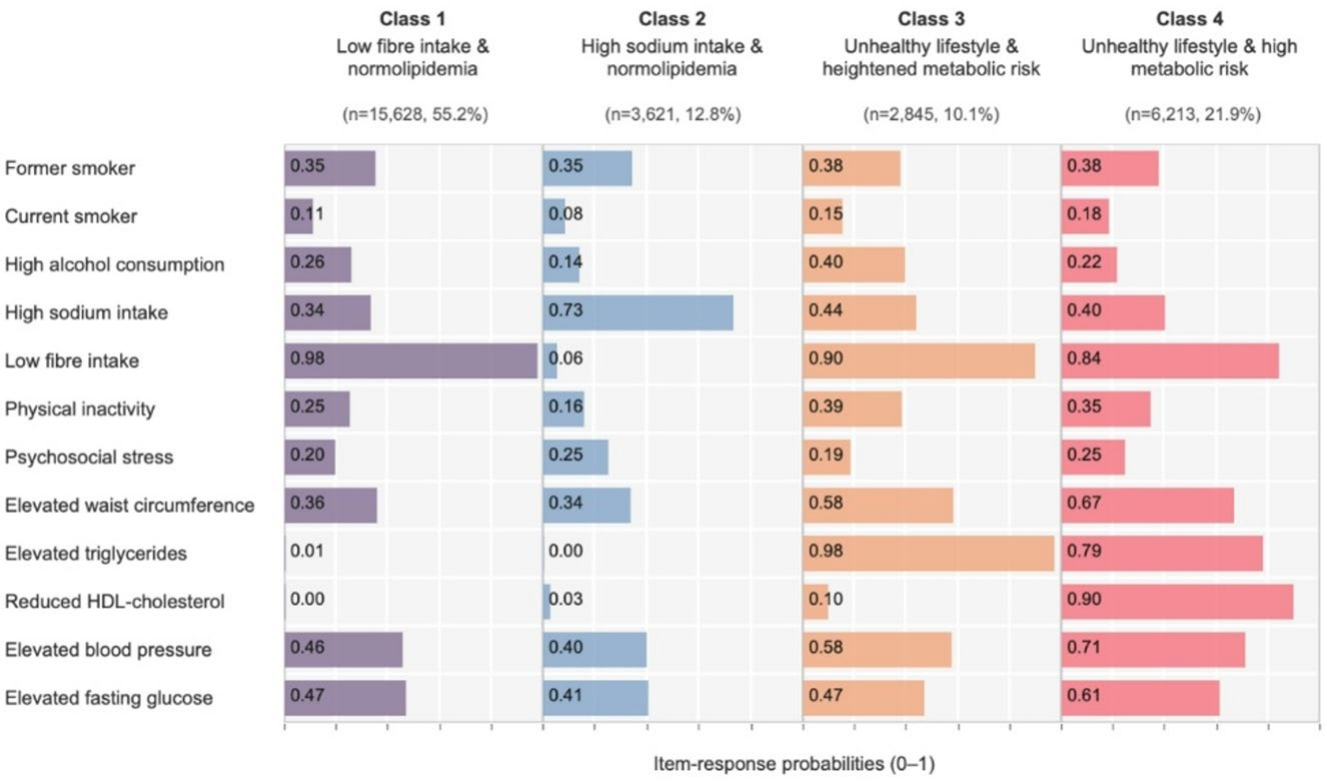
Proportion of participants in each class and the prevalence of risk factors within classes, based on a four-class model of cardiometabolic risk factors in middle-aged Swedish adults (*N* = 28,307).

Class 1, “low fibre intake and normolipidemia” (55.2%), was characterised by a high prevalence of low fibre intake and lipid levels within the normal range. Class 2, “high sodium intake and normolipidemia” (12.8%), included individuals who were more likely to have high sodium intake but normal fibre intake, and had a metabolic profile similar to Class 1. Class 3, “unhealthy lifestyle and heightened metabolic risk” (10.1%), included individuals with an increased probability of adverse lifestyle, particularly high alcohol consumption and physical inactivity. This class also showed an elevated probability of having metabolic abnormalities, especially elevated triglycerides and waist circumference. Class 4, “unhealthy lifestyle and high metabolic risk” (21.9%), showed clustering of behavioural and metabolic risk factors, notably a high probability of reduced HDL-cholesterol (Fig. 2).

Table S5 summarises participant characteristics of the four latent classes. Sex distribution varied across classes: “low fibre intake and normolipidemia” and “unhealthy lifestyle and high metabolic risk” classes had a nearly balanced distribution of men and women, “high sodium intake and normolipidemia” consisted mostly of women (79.7%), while “unhealthy lifestyle and heightened metabolic risk” was predominantly male (73.0%). The “unhealthy lifestyle and heightened metabolic risk” and “unhealthy lifestyle and high metabolic risk” had a greater proportion of individuals who did not attain tertiary education, 59.4% and 59.8% respectively. The employment rate was high in the “low fibre intake and normolipidemia” (88.5%) and high sodium intake and normolipidemia” (85.8%) classes, but lower in “unhealthy lifestyle and heightened metabolic risk” (84.2%) and “unhealthy lifestyle and high metabolic risk” (77.7%) classes.

In addition, we compared the class membership with the number of ATP III components (Table S6) for defining MetS. Among individuals in the “unhealthy lifestyle and high metabolic risk” class, 87.7% had MetS (≥ 3 components). However, MetS was also present in 58.4% of individuals in the “unhealthy lifestyle and heightened metabolic risk” class and 12.6% in the “low fibre intake and normolipidaemia” class.

### Cardiometabolic risk factors classes and subclinical atherosclerosis

The prevalence of subclinical atherosclerosis varied between latent classes (Table 2). For coronary atherosclerosis, the predicted prevalence of having a CAC score ≥ 100 was almost similar in “low fibre intake & normolipidemia” (9.4%, 95% CI 9.0–9.8) and “high sodium intake & normolipidemia” classes (9.3%, 95% CI 8.7–9.9), but higher in “unhealthy lifestyle and heightened metabolic risk” (12.1%, 95% CI 11.1–13.0) and “unhealthy lifestyle and high metabolic risk” (18.0%, 95% CI 17.2–18.9). Based on zero-inflated regression models, the predicted mean CAC scores were 43.0 (95% CI 40.5–45.5) in “low fibre intake and normolipidemia”, 42.6 (95% CI 39.0–46.3) in “high sodium intake and normolipidemia”, and 53.1 (95% CI 48.4–57.8) in “unhealthy lifestyle and heightened metabolic risk”. “Unhealthy lifestyle and high metabolic risk” showed a markedly higher mean CAC score of 92.1 (95% CI, 86.2–98.0). For carotid atherosclerosis, the prevalence of bilateral plaque ranged from 21.5% (95% CI 20.7–22.4) in “high sodium intake & normolipidemia” to 30.6% (95% CI 29.5–31.6) in “unhealthy lifestyle and high metabolic risk”, while the prevalence of unilateral plaques was similar across classes (29.9% to 30.2%). Discrimination of the LCA model was poor for carotid atherosclerosis (bilateral plaques, AUC 0.64, 95% CI 0.63–0.64) and acceptable for coronary atherosclerosis (CACS ≥ 100, AUC 0.73, 95% CI 0.72–0.73). The full results of the full regression models (Table S7–S8), unadjusted results (Table S9), and model fit (Table S10–S13) are available in the Supplementary materials.

**Table 2:** The predicted prevalence of coronary and carotid atherosclerosis and the predicted mean CAC score across latent classes in middle-aged Swedish adults (*N* = 28,307).

Latent classes	Coronary atherosclerosis		Carotid atherosclerosis		
	CAC score^b^	CAC score ≥ 100^a^	Any plaques^a^	Unilateral plaque^c^	Bilateral plaques^c^
	Mean (95% CI)	Prevalence (95% CI)	Prevalence (95% CI)	Prevalence (95% CI)	Prevalence (95% CI)
Class 1: Low fibre intake & normolipidemia	43.0 (40.5–45.5)	9.4 (9.0–9.8)	52.2 (51.4–52.9)	29.9 (29.2–30.6)	22.3 (21.7–22.9)
Class 2: High sodium intake & normolipidemia	42.6 (39.0–46.3)	9.3 (8.7–9.9)	51.6 (50.6–52.6)	30.0 (29.0–30.9)	21.5 (20.7–22.4)
Class 3: Unhealthy lifestyle & heightened metabolic risk	53.1 (48.4–57.8)	12.1 (11.1–13.0)	57.1 (55.5–58.6)	29.9 (28.4–31.4)	27.1 (25.8–28.5)
Class 4: Unhealthy lifestyle & high metabolic risk	92.1 (86.2–98.0)	18.0 (17.2–18.9)	60.8 (59.8–61.9)	30.2 (29.2–31.2)	30.6 (29.5–31.6)

CI: confidence interval; CAC: coronary artery calcification. ^a^ Predicted prevalence of coronary and carotid atherosclerosis was obtained from logistic regression, adjusted for covariates (age, sex, marital status, country of birth, family history of diabetes/CVD, education, employment status, and study site). ^b^ Predicted CAC score was obtained from zero-inflated negative binomial regression, adjusted by covariates. ^c^ Predicted prevalence was obtained from multinomial logistic regression, adjusted by covariates. Full results are available in Table S7–S8.

Standardised mean differences (Cohen’s d) in CAC scores between classes ranged from − 0.003 to 0.27, with the largest difference observed between the “low fibre intake & normolipidemia” and “unhealthy lifestyle & high metabolic risk” classes (Table S14). Marginal differences in bilateral carotid plaque were small, and no notable differences were observed for unilateral plaque across classes (Table S15).

### Sensitivity analysis

Four sensitivity analyses were conducted. *First*, in sex-stratified analyses, a three-class model was identified as the optimum number of classes for females and males, compared with the four-class model in the main analysis (Table S3). The “high sodium intake and normolipidemia” class observed in the pooled model did not persist after stratification (Figures S2). Among females, an intermediate-risk class was characterised predominantly by obesity (“unhealthy lifestyle and obesity”), while among males, the corresponding class combined obesity with hypertriglyceridemia (“unhealthy lifestyle, obesity, hypertriglyceridemia”). However, the stratified results should be interpreted with caution. Entropy was relatively low for both females (0.63) and males (0.61; Table S3). The class-specific mean posterior membership probabilities were low for females in the “unhealthy lifestyle, obesity, hypertriglyceridemia” class (0.37) and for males in the “unhealthy lifestyle and obesity” class (0.34; Tables S16–17), indicating poor class separation. *Second*, replacing triglycerides with LDL-cholesterol in the model did not improve model fit. Both BIC and AIC values were higher compared to the main model, and the entropy value was lower (0.73) (Table S3). *Third*, results from the mixture model were generally consistent with the main (binary) model. The main difference was that the high sodium intake class showed slightly higher metabolic risk in the mixture model. However, this solution had lower entropy and greater classification uncertainty (Figure S3, Table S3, Table S18). *Fourth*, tests for the interaction effect between latent class membership and sex were not statistically significant, suggesting that the associations were adequately captured by an additive effect (Table S19–20).

## Discussion

This study applied a person-centred analytical approach to uncover how cardiometabolic risk factors cluster within individuals and how these clusters associate with subclinical coronary and carotid atherosclerosis using a large, population-based sample in Sweden. We identified distinct classes of risk profiles that extended beyond the traditional assessment of single factors in isolation. Classes 1 and 2 were predominantly shaped by dietary pattern, including “low fibre intake and normolipidemia” and “high sodium intake and normolipidemia”. Class 3 reflected the co-occurrence of adverse lifestyle behaviours with emerging metabolic risk (“unhealthy lifestyle and heightened metabolic risk”). Class 4 showed the clustering of multiple behavioural and high metabolic risks (“unhealthy lifestyle and high metabolic risk”), which has the highest burden of subclinical atherosclerosis. While statistically significant differences in coronary atherosclerosis were observed for certain classes, the absolute differences were modest, and mean CAC scores remained below established clinical thresholds for high cardiovascular risk (> 300).^36^ Carotid plaque prevalence was broadly similar across classes, suggesting limited differentiation of carotid atherosclerosis by these profiles in this cohort.

The latent classes identified in this study have several implications. First, despite having lipid levels within normal range, about half of the individuals in “low fibre intake " and “high sodium intake " classes had carotid artery plaque, and almost one in ten had a CAC score ≥ 100. Notably, the class with high sodium intake also had high fibre intake, with women comprising approximately 80% of this class. While the combination of high sodium and high fibre intake may seem counterintuitive, it is plausible within the Swedish dietary context.^37^ Most sodium consumption comes from processed foods, such as whole-grain rye breads, processed soups, and pickled products, which often contain added salt for flavour, preservation, or leavening, although they may also be fibre-rich.^38,39^ These dietary-pattern classes may help inform dietary counselling and population prevention strategies, including improving food label literacy to help people better identify hidden source of sodium and reducing sodium in staple processed foods often perceived as ‘healthy’, such as certain breads and soups.

Second, the “unhealthy lifestyle & heightened metabolic risk” class represents a distinct class characterised by elevated prevalence of smoking, physical inactivity, and high alcohol consumption. Its co-occurrence with metabolic profiles, including elevated triglycerides and increased waist circumference, aligns with previous evidence linking these behaviours to adverse metabolic outcomes. High alcohol intake has been shown to be a risk of hypertriglyceridemia,^40^ an association likely exacerbated by sedentary behaviour and unhealthy dietary patterns. This pattern suggests that multicomponent lifestyle approaches are a plausible priority for prevention efforts in individuals with similar co-occurrence of cardiometabolic risk factors, while clinical management should still follow guideline-based assessment and targets.^41^ Finally, the “unhealthy lifestyle and high metabolic risk” class may need more intensive risk factor modification in people with high cardiometabolic risk.

Our findings provide population-level patterns of co-occurring cardiometabolic risk factors. The latent classes should be interpreted as statistical profiles rather than clinical phenotypes for individualised care, and they are not a replacement for guideline-based risk assessment or risk stratification tools. Instead, they complement these approaches by highlighting which risk factors tend to co-occur and may help inform prevention planning and counselling. Risk stratification tools identify who is at higher risk, whereas LCA can indicate which modifiable factors commonly cluster within individuals. In the “unhealthy lifestyle and heightened metabolic risk” class, for instance, very high triglycerides co-occurred with elevated alcohol consumption and physical inactivity. While triglycerides should still be managed according to guidelines, this pattern suggests that alcohol intake and physical inactivity may be relevant co-occurring factors to address, rather than treating raised triglycerides as an isolated factor.

Our findings also help contextualise the mixed evidence on multi-factorial lifestyle interventions. A Cochrane review of randomised controlled trials reported that interventions targeting multiple risk factors did not reduce CVD mortality in the general population, but may offer benefits for individuals at higher overall risk.^42^ One possible explanation is that the general population comprises heterogeneous risk profiles; thus, one-size-fits-all multicomponent programmes may be poorly matched to some profiles.

The notable strengths of our study are that it used a large population-based dataset and integrated behavioural, psychosocial, and metabolic risk factors within the LCA models to consider the potential co-occurrence of risk factors. Applying LCA in this study enabled the identification of these nuanced profiles, providing insights that may be missed in traditional variable-based analyses.^15^ Additionally, the SCAPIS study is linked to register data and contains extensive imaging data on subclinical atherosclerosis, rich biomarker testing, and accelerometer data, strengthening our findings.

Nevertheless, this study is subject to limitations. First, risk factors and subclinical atherosclerosis were assessed concurrently due to the cross-sectional design. Consequently, temporality cannot be established, and reverse causation cannot be excluded. To mitigate this concern, we excluded participants with a documented history of coronary heart disease, stroke, or prior revascularisation (PCI or CABG), reducing the likelihood that behaviours had been modified following a clinical diagnosis. Nevertheless, subclinical or undiagnosed disease may still influence certain exposures. A prospective observational study suggests a bidirectional relationship between arterial stiffness, a key vascular change closely associated with subclinical atherosclerosis, and incident hypertension.^43^ Thus, longitudinal studies are needed to clarify temporality. Second, while latent transition analysis could better capture dynamic changes in risk factors, its application was not feasible because SCAPIS had only a single wave of data at the time this manuscript was written. Future research may consider latent transition analysis with longitudinal data, where possible. Third, participation in SCAPIS was lower among individuals with lower socioeconomic status and non-Western backgrounds, ^44^ and participants more often resided in urban areas. While this may affect external validity, previous research indicates that SCAPIS participants were generally comparable to the target population across most cardiometabolic risk factors.^44^ Some differences were observed, including more frequent alcohol consumption, lower smoking prevalence, and slightly lower body mass index.^44^ Although inverse probability weighting is often used to address selection bias, it was not feasible here due to the unavailability of sample weights or individual-level data on non-participants. This limitation may have implications for equity, as it could result in the underrepresentation of groups with greater vulnerability to cardiometabolic disease. Fourth, coronary atherosclerosis was assessed using calcified plaque only (for example, coronary artery calcium), which does not capture non-calcified plaque or high-risk plaque characteristics. This may underestimate the total atherosclerotic burden. Lastly, while we tried to minimise the classification bias by applying a bias-adjusted three-step analysis, the “naming fallacy” (incorrectly labelling or interpreting classes) cannot be entirely eliminated.^14,31^.

In conclusion, latent class analysis identified distinct patterns of co-occurring cardiovascular risk factors. The high metabolic risk class showed a higher burden of coronary atherosclerosis, while differences in carotid atherosclerosis were small. These classes provide a complementary, population-level description of risk factor clustering and may inform prevention planning.

## Supplementary Information

Below is the link to the electronic supplementary material.

Supplementary Material 1

## Data Availability

Data underlying this study cannot be shared publicly due to the privacy of individuals who participated in the study. However, all data used in this study are available from SCAPIS (www.scapis.org) for researchers, subject to applicable policies and ethical approval.
