# Supplementary material for: A latent class analysis of cardiometabolic risk factors and the predicted prevalence of subclinical atherosclerosis in middle-aged Swedish adults
Source: Sci Rep. 2026 Mar 4;16:8255. doi: 10.1038/s41598-026-42858-5 (PMC12963393; doi:10.1038/s41598-026-42858-5)
Supplement: Supplementary file 1 — Supplementary Material 1 [file 41598_2026_42858_MOESM1_ESM.pdf]

Supplementary material

Table S1. List of variables

Variables	Original variables: description	Variables' measurement
**Outcomes**		
Coronary artery calcification (CAC) score	CACS_Tot: Summed CACS (Coronary artery calcium score according to Agatston).<br><br>The sum of the 3 individual CACS scores for the three coronary artery.	Adaptation from Östrogen et al (2021) on Systematic Coronary Risk Evaluation estimated risk and prevalent subclinical atherosclerosis in coronary and carotid artery: A population-based cohort analysis from the Swedish Cardiopulmonary Bioimage Study<br><br>Continuous (score ranged from 0–1045)<br><br>Binary<br>0: No/low (CAC score 1–99)<br>1: Moderate/high (CAC score ≥100)
Carotid artery plaque	carotidplaque: Presence of any carotid artery plaque<br><br>Based on initial on-site analysis of carotid ultrasound imaging of the carotid artery.	0: No plaque (no plaque found in either carotid artery)<br>1: Unilateral plaques (plaque found in one carotid artery)<br>2: Bilateral plaques (plaque found in both carotid artery)
**Cardiometabolic risk factors (independent variables)**		
Smoking	derived_smoke_status: Derived variable based on current smoking status and information on smoking status at biological sampling.	0: Non-smokers<br>1: Former smokers<br>2: Current smokers
Alcohol consumption (sensitivity analysis)	cqah001: Frequency having an alcoholic drink, last year: (0) Never = 0/week; (1) Once a month = 0.25/week; (2) 2–4 times/month = 0.75/week; (3) 2–	Adaptation from the 2021 ESC Guidelines on cardiovascular disease prevention in clinical

Variables	Original variables: description	Variables' measurement
	3 times/week = 2.5/week; (4) ≥ 4 times per week = 4/week.<br>cqah002: Number of alcoholic drinks on a typical drinking day year: (0) 1–2 = 1.5 unit; (1) 3–4 = 3.5 unit; (2) 5–6 = 5.5 unit; (3) 7–9 = 8 unit; (4) ≥10 = 10 unit<br><br>cqah003: Frequency having >6 alcoholic drinks on one occasion: 0) Never; (1) <1 per month; (2) Every month; (3) Every week; (4) Daily/almost everyday	practice (upper limit of 100 g per week) and Wood, et al (2018) (upper limit of 5–6 standard glasses of wine or pints of beer per week).<br><br>The first two AUDIT questions were converted to obtain the frequency and amount of weekly consumption (frequency × number of drinks per day).<br>0: Abstinence/light drinker (score ≤ 6)<br>1: Moderate/heavy drinker (score > 6) or binge drinker (cqah003 ≥ 2)
Sodium intake	natrium: calculated nutrition value, sodium (milligram/mg)	Nordic Nutrition Recommendations 2023.<br>0: ≤2.3 gram/day<br>1: >2.3 gram/day (low fibre intake)
Fibre intake	fibre: calculated nutrition value, fibre (g)	Nordic Nutrition Recommendations 2023.<br>0: ≥25 grams/day for females and ≥35 grams/day for males<br>0: <25 grams/day for females and <35 grams/day for males
Physical activity	validdays: number of valid days of accelerometer recording<br>weartimedaily: Accelerometer wear time (minutes per day)<br>mpa: Moderate intensity physical activity (MPA), total minutes<br>vpa: Vigorous-intensity physical activity (VPA), total minutes<br>seddaily: Sedentary (SED), average minutes per day	WHO guidelines of physical activity: (low VPA <75 min/week and low MPA<150 min/week). High daily sedentary was defined as ≥9.5 hours/day.<br><br>For participants with less than 7 days of valid data (validdays<7), the daily threshold used was 10.7 for 75 min/week, 21.4 for 150 min/week, and 42.8 min for 300 min/week.

Variables	Original variables: description	Variables' measurement
		0: Low risk<br>1: At-risk (highly sedentary [≥9.5 hours/day] AND low VPA [<150 min/week])
Psychosocial stress	cqsle001: By stress we mean feeling tense, irritable, anxious or having sleeping difficulties as a result of conditions at work or at home. Did you experience this?	Adaptation from Rosengen, et al. (2004).<br><br>0: No/several period (Never experienced stress/have experienced a period of stress/some stressful period in the last five years)<br>1: Permanent stress (Consistent stress in the past year/constant stress in the last five years)
Elevated waist circumference	Waist: Waist circumference	The Third Report of the National Cholesterol Education Program Expert Panel on Detection, Evaluation, and Treatment of High Blood Cholesterol in Adults (NCEP ATP III).<br><br>0: No<br>1: Yes (>102 cm in males & >88 cm in females)
Elevated triglycerides	TgFormattedResult: TG Numerical result – national variable.<br><br>Prescribed drug register, ATC code: C10*<br>*Reference: National Board of Health and Welfare. Statistics on pharmaceuticals 2022. 2023. Available at: https://www.socialstyrelsen.se/globalassets/sharepoint-dokument/artikelkatalog/3xcept3ic/2023-3-8457.pdf	NCEP ATP III<br><br>0: No<br>1: Yes (≥150 mg/dl or had been prescribed by lipid lowering drugs in the last 60 months)
Reduced HDL cholesterol	HdlFormattedResult: HDL Numerical result – national variable	NCEP ATP III.<br><br>0: No<br>1: Yes (<1.0 mmol/L in males and <1.3 mmol/L in females or had been prescribed by lipid

Variables	Original variables: description	Variables' measurement
		lowering drugs in the last 60 months)
Elevated LDL-cholesterol (for senstivity analysis)	LdlFormattedResult: LDL Numerical result – national variable	NCEP ATP III<br>0: No<br>1: Yes (≥130 mg/dl or 3.4 mmol/L, borderline high to very high or had been prescribed by lipid lowering drugs in the last 60 months)
Elevated blood pressure	SBP_Mean: Systolic blood pressure, mean brachial (recommended for general analysis).<br><br>Prescribed drug register, ATC codes: C02, C03, C07, C08, C09, excluding C02AC02 and C07AA07*<br>*Reference: National Board of Health and Welfare. Statistics on pharmaceuticals 2022. 2023. Available at: https://www.socialstyrelsen.se/globalassets/sharepoint-dokument/artikelkatalog/4xcept4ic/2023-3-8457.pdf	NCEP ATP III.<br><br>0: No<br>1: Yes (systolic≥ 130 or diastolic ≥ 85 mmHg or had been prescribed by antihypertensive drugs in the last 60 months)
Elevated fasting glucose	Pgluc_Cap: Glucose, capillary, day 1. Capillary plasma glucose from Day 1 performed at each site using fresh blood samples after an over-night fast. Some subjects who had not been fasting prior to biological sampling.<br><br>Prescribed drug register, ATC codes: A10B*<br>*Reference: Hagman E, Danielsson P, Brandt L, Ekbom A, Marcus C. Association between impaired fasting glycaemia in pediatric obesity and type 2 diabetes in young adulthood. Nutr Diabetes. 2016 Aug 22;6(8):e227. Doi: 10.1038/nutd.2016.34. PMID: 27548712; PMCID: PMC5022148.	NCEP ATP III.<br><br>0: No<br>1: Yes (≥ 110 mg/dl or had been prescribed by blood glucose lowering drugs in the last 60 months)
**Covariates**		
Age	AgeAtVisitOne: Age at study visit 1, rounded to 1 decimal	0: 50–59 years<br>1: 60–64 years
Sex	Sex	0: Female<br>1: Male
Marital status	cqli003: Current marital status	0: Married (registered partnership/cohabitating)<br>1: Single (unmarried/living alone)<br>2: Divorce/widow
Country of birth	cqli009: Born in Sweden	0: Sweden (Swedish-born)

Variables	Original variables: description	Variables' measurement
		1: Outside Sweden (Foreign-bron)
Parental history of cardiometabolic disease	cqdf002: Family history of diabetes, subject's mother<br>cqdf003: Family history of diabetes, subject's father<br>cqdf017: Family history of myocardial infarction, subject's mother<br>cqdf018: Family history of myocardial infarction, subject's father<br>cqdf022: Family history of stroke, subject's father<br>cqdf023: Family history of stroke, subject's mother	0: No (if respondents answered "no" for family history of diabetes, myocardial infarction, and stroke)<br>1: Yes (if respondents had family history of diabetes, myocardial infarction and stroke from mother and/or father)
Educational attainment	cqed001: Highest completed level of education	0: Tertiary (university/college)<br>1: Secondary (upper secondary, higher secondary, vocational or equivalen)<br>2: Primary or lower (did not complete primary or other compulsory education, lower secondary or equivalent (maximum 9 years))
Employment status	cqed014: Professional work, last 12 months	0: Unemployed<br>1: Employed
Site	Site: Site of examination	0: Gothenburg<br>1: Linköping<br>2: Malmö<br>3: Stockholm<br>4: Umeå<br>5: Uppsala

Table S2. Percentages of missing data

Variable	Missing (n)	%
**All SCAPIS participants**	**30154**	
Exclusion criteria (see Figure S1)	1597	
Excluded due to missing values in outcome variables	250	
**Total sample for main analysis**	**28307**	**100**
Missing values in independent variables	4744	16.8
**Variables with missing value:**		
Smoking	831	3.2
Alcohol consumption	792	3.2
Sodium intake	556	2
Fibre intake	556	2
Physical activity	1954	7.5
Psychosocial stress	1031	4.2
Waist circumference	11	0.1
Triglycerides	72	0.2
HDL-cholesterol	65	0.3
Blood pressure	5	0.3
Fasting glucose	30	0.1
Age	0	0
Sex	0	0
Marital status	743	3
Family history of CVD	1008	4
Education	697	2.8
Employment	796	3.2
Financial difficulty	840	3.4
Country of birth	668	2.7
Residency (DeSO)	130	0.5

Table S3. Latent class analysis fit statistics

Classes	Log-likelihood	BIC	AIC	SABIC	CAIC	VLMR	Entropy
**Main analysis: all respondents**							
1	-188531.42	377544.63	377156.84	377395.27	377591.63	N/A	1
2	-182780.79	366176.63	365681.57	365985.95	366236.63	<0.0001	0.76
3	-182648.41	366045.14	365442.82	365813.15	366118.14	<0.0001	0.85
**4**	**-182577.05**	**366035.66**	**365326.09**	**365762.36**	**366121.66**	**<0.0001**	**0.81**
5	-182532.03	366078.90	365262.06	365764.28	366177.90	<0.0001	0.55
6	-182507.08	366162.25	365238.15	365806.32	366274.25	0.049	0.51
**Stratified analysis: females**							
1	-95972.51	192396.12	192039.03	192277.46	192443.12	N/A	1
2	-92788.85	186153.56	185697.69	186002.07	186213.56	<0.0001	0.79
**3**	**-92720.05**	**186140.73**	**185586.10**	**185956.42**	**186213.73**	**<0.0001**	**0.63**
4	-92687.18	186199.77	185546.36	185982.63	186285.77	<0.0001	0.51
5	-92664.93	186280.03	185527.85	186030.07	186379.03	0.003	0.52
6	-92640.05	186355.06	185504.11	186072.27	186467.06	0.031	0.44
**Stratified analysis: males**							
1	-88767.17	177981.60	177628.35	177866.77	178028.60	N/A	1
2	-85803.28	172177.52	171726.56	172030.93	172237.52	<0.0001	0.79
**3**	**-85748.66**	**172191.99**	**171643.32**	**172013.65**	**172264.99**	**<0.0001**	**0.61**
4	-85730.31	172278.99	171632.62	172068.89	172364.99	0.005	0.52
5	-85712.91	172367.91	171623.83	172126.05	172466.91	0.359	0.48
6	-85692.07	172449.93	171608.14	172176.31	172561.93	0.078	0.56
**Sensitivity analysis: LDL-c replaced triglycerides**							
1	-190442.63	381377.29	380981.25	381224.75	381425.29	N/A	1
2	-189304.51	379234.33	378731.03	379040.48	379295.33	<0.0001	0.63
**3**	**-189192.33**	**379143.23**	**378532.67**	**378908.06**	**379217.23**	**<0.0001**	**0.65**
4	-189134.02	379159.86	378442.03	378883.37	379246.86	<0.0001	0.61
5	-189090.56	379206.21	378381.13	378888.42	379306.21	0.050	0.50
6	-189087.25	379332.85	378400.50	378973.74	379445.85	0.278	0.44
**Sensitivity analysis: Mixture model**							
1	-235742.66	471628.84	471513.33	471584.35	471642.84	N/A	1
2	-219256.05	438809.37	438570.10	438717.21	438838.37	<0.0001	0.6954
3	-212226.95	424904.95	424541.91	424765.12	424948.95	<0.0001	0.6793
**4**	**-207072.35**	**414749.51**	**414262.71**	**414562.01**	**414808.51**	**<0.0001**	**0.7188**
5	-202562.80	405884.17	405273.61	405649.00	405958.17	<0.0001	0.7330
6	-201854.56	404621.45	403887.12	404338.61	404710.45	<0.0001	0.7296

Notes:

AIC: Akaike Information Criterion, BIC: Bayesian Information Criterion, CAIC: consistent Akaike Information Criterion, SABIC: sample size Adjusted Bayesian Information Criterion, VLMR: Vuong-Lo-Mendell-Rubin Test.

Lower values of AIC, CAIC, BIC, and SABIC and higher entropy indicated better model fit.

The selected optimum number of classes is shown in bold.

In the mixture model, the 4-class solution was selected, as the 5- and 6-class models included classes with very small proportions (<5%).

Table S4. Misclassification matrix: all respondents

			The assigned class membership			
			**Class 1**	**Class 2**	**Class 3**	**Class 4**
			Low fibre intake & normolipidemia	High sodium intake & normolipidemia	Unhealthy lifestyle & heightened metabolic risk	Unhealthy lifestyle & high metabolic risk
**Latent class**	**Class 1**	Low fibre intake & normolipidemia	**0.9508**	0.0334	0.0070	0.0089
	**Class 2**	High sodium intake & normolipidemia	0.1446	**0.8271**	0.0024	0.0260
	**Class 3**	Unhealthy lifestyle & heightened metabolic risk	0.0424	0.0034	**0.7099**	0.2443
	**Class 4**	Unhealthy lifestyle & high metabolic risk	0.0211	0.0142	0.0954	**0.8693**

Table S5. Participants' characteristics by latent class

Variables	Class 1<br>Low fibre intake & normolipidemia	Class 2<br>High sodium intake & normolipidemia	Class 3<br>Unhealthy lifestyle & heightened metabolic risk	Class 4<br>Unhealthy lifestyle & high metabolic risk
N (%[a])	15,628 (55.2)	3,621 (12.8)	2,845 (10.1)	6,213 (21.9)
Age, mean (SD)	56.7 (4.3)	57.2 (4.3)	56.8 (4.3)	57.8 (4.3)
Sex				
Female	7,721 (49.4)	2,886 (79.7)	767 (27.0)	3,358 (54.0)
Male	7,907 (50.6)	735 (20.3)	2,078 (73.0)	2,855 (46.0)
Marital status				
Single	1,909 (12.2)	488 (13.5)	386 (13.6)	911 (14.7)
Married	11,575 (74.1)	2,497 (69.0)	2,048 (72.0)	4,213 (67.8)
Divorce/widow	1,803 (11.5)	543 (15.0)	320 (11.2)	872 (14.0)
Missing	341 (2.2)	93 (2.6)	91 (3.2)	217 (3.5)
Country of birth				
Swedish-born	13,357 (85.5)	2,866 (79.1)	2,288 (80.4)	4,737 (76.2)
Foreign-born	1,962 (12.6)	677 (18.7)	478 (16.8)	1,274 (20.5)
Missing	309 (2.0)	78 (2.2)	79 (2.8)	202 (3.3)
Parental history of CVD				
No	7,463 (47.8)	1,635 (45.2)	1,320 (46.4)	2,319 (37.3)
Yes	7,688 (49.2)	1,858 (51.3)	1,410 (49.6)	3,606 (58.0)
Missing	477 (3.1)	128 (3.5)	115 (4.0)	288 (4.6)
Education				
Tertiary	7,299 (46.7)	1,929 (53.3)	1,066 (37.5)	2,295 (36.9)
Secondary	6,794 (43.5)	1,367 (37.8)	1,422 (50.0)	2,956 (47.6)
Primary or lower	1,218 (7.8)	240 (6.6)	268 (9.4)	756 (12.2)
Missing	317 (2.0)	85 (2.3)	89 (3.1)	206 (3.3)
Employment status				
Employed	13,827 (88.5)	3,106 (85.8)	2,396 (84.2)	4,827 (77.7)
Unemployed	1,437 (9.2)	419 (11.6)	352 (12.4)	1,147 (18.5)
Missing	364 (2.3)	96 (2.7)	97 (3.4)	239 (3.8)
Site				
Göteborg	3,250 (20.8)	814 (22.5)	582 (20.5)	1,257 (20.2)
Linköping	2,847 (18.2)	540 (14.9)	476 (16.7)	928 (14.9)
Malmö	3,070 (19.6)	749 (20.7)	637 (22.4)	1,364 (22.0)
Stockholm	2,808 (18.0)	594 (16.4)	359 (12.6)	952 (15.3)
Umeå	1,216 (7.8)	302 (8.3)	298 (10.5)	551 (8.9)
Uppsala	2,437 (15.6)	622 (17.2)	493 (17.3)	1,161 (18.7)
CAC score				
0	9,926 (63.5)	2,568 (70.9)	1,462 (51.4)	2,894 (46.6)
1–99	4,278 (27.4)	816 (22.5)	985 (34.6)	2,043 (32.9)
100–399	1,034 (6.6)	182 (5.0)	298 (10.5)	806 (13.0)
≥400	390 (2.5)	55 (1.5)	100 (3.5)	470 (7.6)
CAC score, median (IQR)[b]	29 (7–100)	27 (6–89)	36 (9–118)	58 (13–207)
Carotid artery plaques				
No plaque	7,570 (48.4)	1,870 (51.6)	1,131 (39.8)	2,309 (37.2)
Unilateral plaque	4,661 (29.8)	1,061 (29.3)	866 (30.4)	1,870 (30.1)
Bilateral plaque	3,397 (21.7)	690 (19.1)	848 (29.8)	2,034 (32.7)

Table S6. Agreement between the finding of LCA model and the number of NCEP components of the metabolic syndrome definition.

			Number of the NCEP components of MetS			
			**Zero**	**One**	**Two**	**Three or more**
**Latent class**	**Class 1**	Low fibre intake & normolipidemia	3,616 (23.1)	5,732 (36.7)	4,309 (27.6)	1,969 (12.6)
	**Class 2**	High sodium intake & heightened metabolic risk	1,002 (27.7)	1,395 (38.5)	903 (24.9)	321 (8.9)
	**Class 3**	Unhealthy lifestyle & heightened metabolic risk	0 (0.0)	375 (13.2)	805 (28.4)	1,654 (58.4)
	**Class 4**	Unhealthy lifestyle & high metabolic risk	0 (0.0)	108 (1.7)	655 (10.5)	5,463 (87.7)
		**Total**	4,618 (16.3)	7,610 (26.9)	6,672 (23.6)	9,407 (33.2)

Table S7. Association between latent classes and CAC score in Swedish middle-aged adults (N=28,307)

Variables	Logistic regression[a]		Zero-inflated negative binomial			
			Logit part[b]		Negative binomial part[c]	
	OR (95% CI)	*p* value	OR (95% CI)	*p* value	Coef. (95% CI)	*p* value
Latent class (ref. Class 1: low fibre intake & normolipidemia)						
Class 2: High sodium intake & normolipidemia	0.99 (0.92–1.07)	0.843	2.84 (2.67–3.04)	0.164	1.01 (0.92–1.10)	0.897
Class 3: Unhealthy lifestyle & heightened metabolic risk	1.35 (1.22–1.51)	<0.0001	2.07 (1.92–2.25)	<0.0001	1.12 (1.00–1.24)	0.041
Class 4: Unhealthy lifestyle & high metabolic risk	2.29 (2.11–2.49)	<0.0001	1.63 (1.57–1.70)	<0.0001	1.74 (1.60–1.91)	<0.0001
Age	1.14 (1.13–1.15)	<0.0001	2.41 (2.39–2.43)	<0.0001	1.08 (1.07–1.09)	<0.0001
Sex						
Male	4.04 (3.71–4.40)	<0.0001	1.26 (1.24–1.28)	<0.0001	2.05 (1.87–2.25)	<0.0001
Marital status (ref. married)						
Single	1.17 (1.04–1.31)	0.008	2.60 (2.37–2.89)	0.407	1.17 (1.03–1.32)	0.014
Divorce/widow	1.16 (1.03–1.31)	0.017	2.52 (2.30–2.81)	0.163	1.02 (0.90–1.15)	0.746
Country of birth (ref. Swedish-born)						
Foreign-born	1.02 (0.92–1.13)	0.728	2.72 (2.47–3.02)	0.997	1.01 (0.90–1.13)	0.875
Parental history of cardiometabolic disease (ref. no)						
Yes	1.32 (1.22–1.43)	<0.0001	2.18 (2.07–2.32)	<0.0001	1.18 (1.08–1.28)	0.000
Education (ref. tertiary)						
Secondary	1.17 (1.08–1.28)	0.000	2.34 (2.20–2.50)	<0.0001	1.14 (1.05–1.25)	0.003
Primary or lower	1.16 (1.02–1.33)	0.027	2.07 (1.88–2.30)	<0.0001	1.29 (1.09–1.53)	0.003
Employment status (ref. employed)						
Unemployed	1.19 (1.06–1.33)	0.003	2.43 (2.21–2.71)	0.044	1.17 (1.04–1.31)	0.006
Site (ref. Göteborg)						
Linköping	0.99 (0.88–1.13)	0.934	2.69 (2.42–3.04)	0.866	0.94 (0.82–1.07)	0.357
Malmö	1.16 (1.03–1.31)	0.012	2.21 (2.04–2.43)	<0.0001	1.10 (0.97–1.25)	0.127
Stockholm	1.03 (0.90–1.16)	0.694	2.63 (2.37–2.96)	0.571	1.04 (0.90–1.20)	0.565
Umeå	1.09 (0.94–1.27)	0.261	2.40 (2.13–2.76)	0.080	1.02 (0.85–1.22)	0.836
Uppsala	0.88 (0.78–1.00)	0.059	3.35 (2.94–3.87)	0.001	0.95 (0.83–1.10)	0.501

Notes:
CI: confidence interval, CAC: coronary artery calcification, Coef: coefficient, OR: odds ratio
[a] Logistic regression model. The OR represent the odds of having moderate/high CAC score (≥100)
[b] Logit part of zero-inflated negative binomial model. The OR represent the odds of having zero CAC score
[c] Negative binomial part of zero-inflated negative binomial model was used to estimate the CAC score for participants with a CAC score> 0.

Table S8. Association between latent classes and the presence of carotid artery plaque in Swedish middle-aged adults (N=28,307)

Variables	Any carotid plaque		Unilateral plaques		Bilateral plaques	
	OR (95% CI)[a]	*p* value	RRR (95% CI)[b]	*p* value	RRR (95% CI)[b]	*p* value
Latent class (ref. Class 1: low fibre intake & normolipidemia)						
Class 2: High sodium intake & normolipidemia	0.95 (0.90–1.00)	0.072	0.99 (0.94–1.04)	0.661	0.95 (0.90–1.00)	0.072
Class 3: Unhealthy lifestyle & heightened metabolic risk	1.38 (1.26–1.51)	<0.0001	1.13 (1.03–1.23)	0.007	1.38 (1.26–1.51)	<0.0001
Class 4: Unhealthy lifestyle & high metabolic risk	1.73 (1.61–1.85)	<0.0001	1.25 (1.17–1.34)	<0.0001	1.73 (1.61–1.85)	<0.0001
Age	1.11 (1.11–1.12)	<0.0001	1.05 (1.05–1.06)	<0.0001	1.11 (1.11–1.12)	<0.0001
Sex						
Male	2.04 (1.92–2.17)	<0.0001	1.41 (1.33–1.49)	<0.0001	2.04 (1.92–2.17)	<0.0001
Marital status (ref. married)						
Single	1.19 (1.08–1.30)	0.000	0.97 (0.89–1.06)	0.492	1.19 (1.08–1.30)	0.000
Divorce/widow	1.10 (1.00–1.20)	0.059	1.01 (0.92–1.10)	0.887	1.10 (1.00–1.20)	0.059
Country of birth (ref. Swedish-born)						
Foreign-born	1.02 (0.94–1.11)	0.625	0.99 (0.91–1.07)	0.793	1.02 (0.94–1.11)	0.625
Parental history of cardiometabolic disease (ref. no)						
Yes	1.15 (1.08–1.22)	<0.0001	1.02 (0.97–1.08)	0.442	1.15 (1.08–1.22)	<0.0001
Education (ref. tertiary)						
Secondary	1.16 (1.09–1.24)	<0.0001	1.08 (1.02–1.15)	0.008	1.16 (1.09–1.24)	<0.0001
Primary or lower	1.38 (1.24–1.54)	<0.0001	1.00 (0.90–1.12)	0.958	1.38 (1.24–1.54)	<0.0001
Employment status (ref. employed)						
Unemployed	1.00 (0.91–1.10)	0.955	1.00 (0.91–1.09)	0.957	1.00 (0.91–1.10)	0.955
Site (ref. Göteborg)						
Linköping	0.94 (0.86–1.04)	0.255	0.93 (0.85–1.02)	0.108	0.94 (0.86–1.04)	0.255
Malmö	1.24 (1.13–1.36)	<0.0001	1.07 (0.98–1.16)	0.143	1.24 (1.13–1.36)	<0.0001
Stockholm	0.60 (0.54–0.67)	<0.0001	0.74 (0.68–0.81)	<0.0001	0.60 (0.54–0.67)	<0.0001
Umeå	0.60 (0.53–0.68)	<0.0001	0.70 (0.62–0.78)	<0.0001	0.60 (0.53–0.68)	<0.0001
Uppsala	0.92 (0.83–1.01)	0.094	0.98 (0.89–1.07)	0.599	0.92 (0.83–1.01)	0.094

Notes:
CI: confidence interval, RRR: relative risk ratio, OR: odds ratio
Reference group for outcome variable (carotid artery plaque): no plaque.
[a] Predicted prevalence obtained from logistic regression.
[b] Predicted prevalence obtained from multinomial logistic regression.

Table S9. The *unadjusted* predicted prevalence of coronary and carotid atherosclerosis across latent classes in Swedish middle-aged adults (N=28,307)

Latent classes	**Coronary atherosclerosis**		**Carotid atherosclerosis**		
	CAC score[b]	CAC score ≥ 100[a]	Any plaque[a]	Unilateral plaque[c]	Bilateral plaque[c]
	Mean score (95% CI)	Prevalence (95% CI)	Prevalence (95% CI)	Prevalence (95% CI)	Prevalence (95% CI)
Class 1**:** Low fibre intake & normolipidemia	42.2 (39.6–44.8)	9.2 (8.8–9.7)	51.7 (51.0–52.5)	29.8 (29.1–30.5)	21.9 (21.3–22.5)
Class 2: High sodium intake & normolipidemia	31.7 (29.0–34.5)	7.3 (6.8–7.8)	49.1 (48.1–50.2)	29.5 (28.6–30.5)	19.6 (18.8–20.4)
Class 3: Unhealthy lifestyle & heightened metabolic risk	65.8 (59.5–72.1)	14.7 (13.6–15.9)	60.0 (58.4–61.5)	30.3 (28.8–31.8)	29.6 (28.2–31.1)
Class 4: Unhealthy lifestyle & high metabolic risk	108.0 (100.7–115.3)	19.9 (19.0–20.9)	62.6 (61.5–63.7)	30.1 (29.0–31.1)	32.5 (31.5–33.6)

Notes:
CI: confidence interval; CAC: coronary artery calcification; SD: standard deviation
[a] Predicted prevalence obtained from logistic regression.
[b] Predicted CAC score obtained from zero-inflated negative binomial regression.
[c] Predicted prevalence obtained from multinomial logistic regression.

Table S10. Discrimination and calibration diagnostics for binary and multinomial logistic regression models

Models	Outcome	AUC (95% CI)	Calibration slope β (95% CI)	Calibration intercept β (95% CI)
Coronary atherosclerosis	CAC ≥100	0.729 (0.724–0.733)	1.002 (0.961–1.043)	0.003 (−0.077 to 0.082)
Carotid atherosclerosis	Absent (0)	0.623 (0.620–0.626)	1.001 (0.951–1.050)	0.000 (−0.025 to 0.026)
	Unilateral (1)	0.533 (0.529–0.537)	1.001 (0.803–1.199)	0.001 (−0.169 to 0.171)
	Bilateral (2)	0.641 (0.637–0.644)	1.002 (0.952–1.051)	0.002 (−0.057 to 0.060)

Table S11. Discrimination (AUC, 95% CI) of LCA, ATP III and SCORE2 for coronary and carotid atherosclerosis.

Models	Coronary atherosclerosis	Carotid atherosclerosis		
	CACS ≥100	Absent	Unilateral (1)	Bilateral (2)
LCA	0.619 (0.609–0.628)	0.554 (0.548–0.560)	0.504 (0.496–0.510)	0.569 (0.561–0.576)
ATP III	0.638 (0.629–0.647)	0.563 (0.556–0.569)	0.510 (0.503–0.517)	0.579 (0.572–0.586)
SCORE2	0.740 (0.731–0.749)	0.640 (0.634–0.647)	0.526 (0.518–0.533)	0.660 (0.652–0.667)
LCA + cov	0.729 (0.724–0.733)	0.623 (0.620–0.626)	0.533 (0.529–0.537)	0.641 (0.637–0.644)
ATP III + cov	0.762 (0.753–0.770)	0.638 (0.631–0.644)	0.535 (0.528–0.542)	0.661 (0.653–0.668)
SCORE2 + cov	0.747 (0.738–0.755)	0.651 (0.645–0.658)	0.535 (0.528–0.542)	0.673 (0.666–0.681)

Table S12. Information-criterion comparison of count models for continuous CAC scores

Model	AIC	BIC
Poisson	5260133.0	5260306.0
Negative binomial	162179.0	162362.2
Zero-inflated negative binomial	159010.7	159367.2

Table S13. Observed versus ZINB-predicted CAC score distribution to assess distributional fit

CAC score	Observed proportion (95% CI)	Predicted proportion (95% CI)
Pr(CAC = 0)	0.595 (0.594–0.597)	0.596 (0.596–0.597)
Pr(CAC = 1)	0.030 (0.029–0.030)	0.030 (0.030–0.030)
Pr(CAC = 2)	0.016 (0.016–0.016)	0.019 (0.019–0.019)
Pr(CAC = 3)	0.012 (0.012–0.013)	0.014 (0.014–0.014)
Pr(CAC = 4)	0.011 (0.011–0.011)	0.011 (0.011–0.011)
Pr(CAC ≥ 5)	0.336 (0.334–0.337)	0.330 (0.329–0.330)

Table S14. Predicted mean CAC scores, marginal differences, and standardised mean differences (Cohen's d) across latent class

Latent classes	Predicted mean CAC score (SD)	Marginal differences (95% CI)	Cohen's d (95% CI)
Class 1: Low fibre intake & normolipidemia	42.71 (158.50)	Ref.	Ref.
Class 2: High sodium intake & heightened metabolic risk	42.28 (120.35)	0.006 (-0.080 – 0.092)	-0.003 (-0.039–0.033)
Class 3: Unhealthy lifestyle & heightened metabolic risk	53.12 (125.32)	0.110 (0.004 – 0.215)	0.068 (0.028–0.108)
Class 4: Unhealthy lifestyle & high metabolic risk	93.76 (236.27)	0.556 (0.467 – 0.645)	0.277 (0.248–0.307)

Notes: Adjusted means were obtained from predictive margins following multiple-imputation zero-inflated negative binomial regression (ZINB), incorporating sampling weights to account for latent class misclassification.

Table S15. Average marginal differences in predicted probability of carotid plaque (unilateral and bilateral) by latent class

Latent classes	Carotid atherosclerosis	
	**Unilateral plaque**	**Bilateral plaque**
	Marginal differences (95% CI)	Marginal differences (95% CI)
Class 1: Low fibre intake & normolipidemia	Ref.	Ref.
Class 2: High sodium intake & heightened metabolic risk	0.001 (–0.008 – 0.011)	–0.008 (–0.016 – 0.001)
Class 3: Unhealthy lifestyle & heightened metabolic risk	0.000 (–0.016 – 0.017)	0.048 (0.033 – 0.063)
Class 4: Unhealthy lifestyle & high metabolic risk	0.003 (–0.009 – 0.016)	0.082 (0.070 – 0.094)

Notes: Marginal differences were obtained following multinomial logistic regression, using dy/dx.

Table S16. Misclassification matrix: females

			The assigned class membership		
			**Class 1**	**Class 2**	**Class 3**
			Low fibre intake & normolipidemia	Unhealthy lifestyle & obesity	Unhealthy lifestyle & high metabolic risk
**Latent class**	**Class 1**	Low fibre intake & normolipidemia	**0.8988**	0.0754	0.0258
	**Class 2**	Unhealthy lifestyle & obesity	0.3949	**0.3418**	0.2633
	**Class 3**	Unhealthy lifestyle & high metabolic risk	0.0665	0.1298	**0.8037**

Table S17. Misclassification matrix: males

			The assigned class membership (W)		
			**Class 1**	**Class 2**	**Class 3**
			Low fibre intake & normolipidemia	Unhealthy lifestyle, obesity, hypertriglyceridemia	Unhealthy lifestyle & high metabolic risk
**Latent class**	**Class 1**	Low fibre intake & normolipidemia	**0.8761**	0.0931	0.0308
	**Class 2**	Unhealthy lifestyle, obesity, hypertriglyceridemia	0.4772	**0.3677**	0.1551
	**Class 3**	Unhealthy lifestyle & high metabolic risk	0.0796	0.0781	**0.8423**

Table S18. Misclassification matrix: mixture model

			The assigned class membership			
			**Class 1**	**Class 2**	**Class 3**	**Class 4**
			Low fibre intake & normolipidemia	High sodium intake & heightened metabolic risk	Unhealthy lifestyle & heightened metabolic risk	Unhealthy lifestyle & high metabolic risk
**Latent class**	**Class 1**	Low fibre intake & normolipidemia	**0.7911**	0.0356	0.111	0.0623
	**Class 2**	High sodium intake & heightened metabolic risk	0.1035	**0.7741**	0.083	0.0394
	**Class 3**	Unhealthy lifestyle & heightened metabolic risk	0.118	0.0303	**0.7943**	0.0574
	**Class 4**	Unhealthy lifestyle & high metabolic risk	0.1134	0.0247	0.0983	**0.7636**

Table S19. Association between latent classes and CAC score in Swedish middle-aged adults (interaction between latent class membership and sex)

**Variables**	**Logistic regression**		**Zero-inflated negative binomial**			
			**Logit part**		**Negative binomial part**	
	OR (95% CI)	*p* value	OR (95% CI)	*p* value	Coef. (95% CI)	*p* value
Latent class (ref. Class 1: low fibre intake & normolipidemia)						
Class 2: High sodium intake & normolipidemia	0.96 (0.83–1.11)	0.600	2.66 (2.35–3.07)	0.766	1.07 (0.99–1.16)	0.101
Class 3: Unhealthy lifestyle & heightened metabolic risk	1.49 (1.17–1.90)	0.001	2.79 (2.34–3.45)	0.786	0.70 (0.59–0.83)	<0.0001
Class 4: Unhealthy lifestyle & high metabolic risk	2.22 (1.91–2.58)	<0.0001	4.94 (3.87–6.59)	<0.0001	0.53 (0.47–0.59)	<0.0001
Sex (ref. female)						
Male	3.97 (3.50–4.50)	<0.0001	6.97 (5.47–9.19)	<0.0001	0.24 (0.22–0.27)	<0.0001
Latent class x sex (ref. Class 1 * female)						
Class 2 * male	1.05 (0.89–1.24)	0.575	2.80 (2.37–3.41)	0.741	0.95 (0.84–1.07)	0.385
Class 3 * male	0.90 (0.69–1.17)	0.427	3.06 (2.45–4.05)	0.322	1.03 (0.83–1.28)	0.778
Class 4 * male	1.05 (0.87–1.25)	0.623	3.13 (2.56–3.99)	0.181	0.78 (0.65–0.93)	0.007

Notes: Regressions were adjusted by all covariates (age, marital status, country of birth, parental history, education, employment, study site)

Table S20. Association between latent classes and the presence of carotid artery plaque in Swedish middle-aged adults (interaction between latent class membership and sex)

**Variables**	One vessel		Both vessels	
	RRR (95% CI)	*P*-value	RRR (95% CI)	*P*-value
Latent class (ref. Class 1: low fibre intake & normolipidemia)				
Class 2: High sodium intake & normolipidemia	1.01 (0.94–1.07)	0.838	0.95 (0.88–1.03)	0.256
Class 3: Unhealthy lifestyle & heightened metabolic risk	1.01 (0.87–1.16)	0.915	1.46 (1.25–1.70)	<0.0001
Class 4: Unhealthy lifestyle & high metabolic risk	1.25 (1.14–1.36)	<0.0001	1.80 (1.63–1.98)	<0.0001
Sex (ref. female)				
Male	1.40 (1.31–1.51)	<0.0001	2.10 (1.93–2.28)	<0.0001
Latent class x sex (ref. Class 1 * female)				
Class 2 * male	0.94 (0.85–1.03)	0.197	0.99 (0.89–1.11)	0.913
Class 3 * male	1.17 (0.97–1.39)	0.095	0.93 (0.77–1.12)	0.463
Class 4 * male	1.01 (0.88–1.15)	0.899	0.93 (0.81–1.07)	0.311

Notes: Regressions were adjusted by all covariates (age, marital status, country of birth, parental history, education, employment, study site)

Figure S1. Sample flowchart

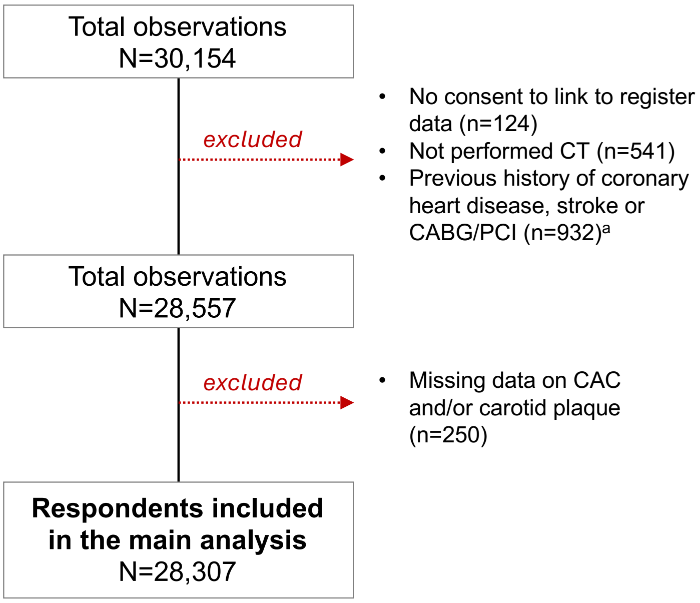

[a] History of coronary heart disease, stroke and percutaneous coronary intervention (PCI) and coronary artery bypass grafting (CABG) were identified based on self-reported information and National Patient Register.

Figure S2. Proportion of participants in each class and the prevalence of risk factors within classes, based on a three-class model of cardiometabolic risk factors in Swedish middle-aged adults, stratified by sex.

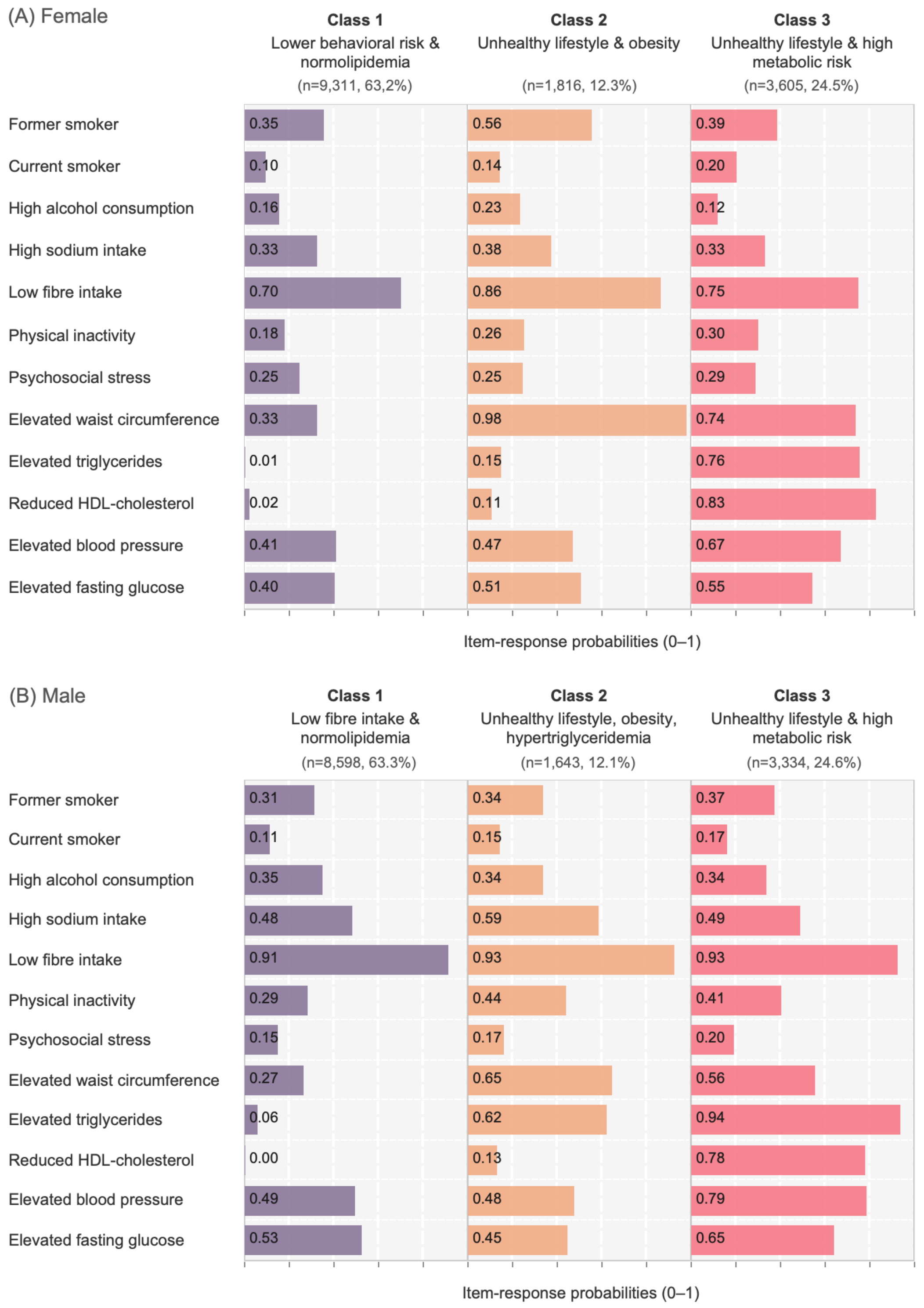

Figure S3. Proportion of participants in each class and the probability/mean of risk factors within classes (mixture model)

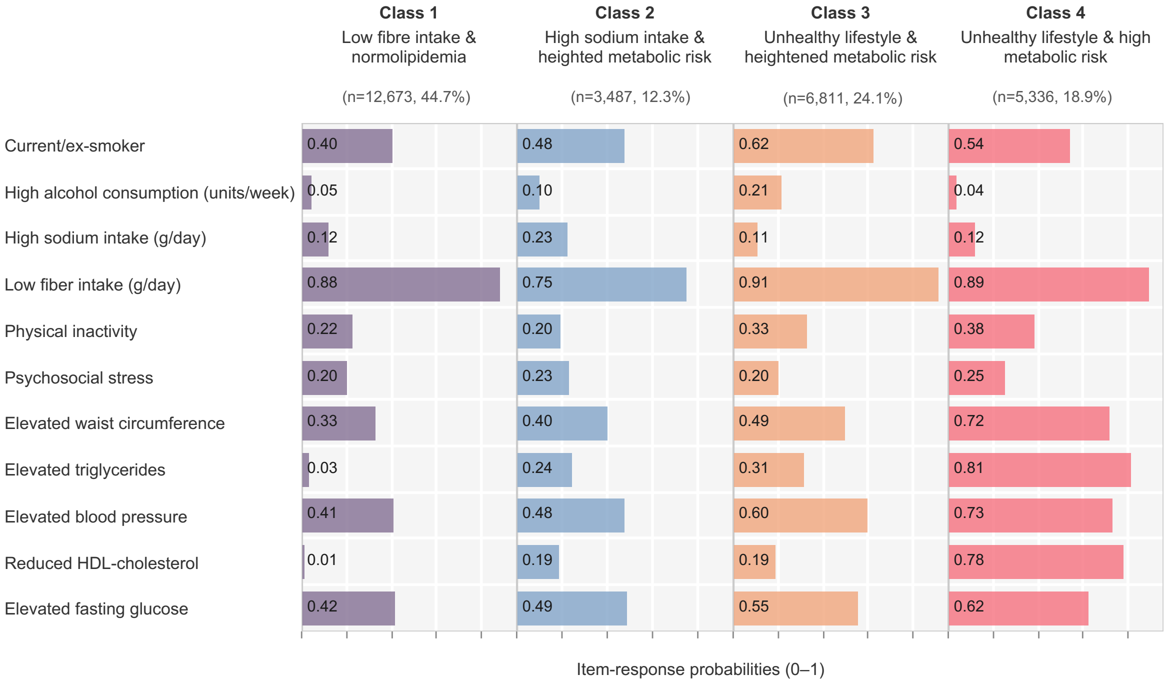

Notes: All continuous variables, alcohol consumption, sodium intake, and fibre intake, were standardised and rescaled to a 0–1 range. Fibre intake was reverse-coded so that higher values consistently reflected greater risk across all indicators.
